# Safety of JAK and IL-6 inhibitors in patients with rheumatoid arthritis: a multicenter cohort study

**DOI:** 10.3389/fimmu.2023.1267749

**Published:** 2023-10-02

**Authors:** Shuhei Yoshida, Masayuki Miyata, Eiji Suzuki, Takashi Kanno, Yuya Sumichika, Kenji Saito, Haruki Matsumoto, Jumpei Temmoku, Yuya Fujita, Naoki Matsuoka, Tomoyuki Asano, Shuzo Sato, Kiyoshi Migita

**Affiliations:** ^1^ Department of Rheumatology, Fukushima Medical University School of Medicine, Fukushima, Japan; ^2^ Department of Rheumatology, Japanese Red Cross Fukushima Hospital, Fukushima, Japan; ^3^ Department of Rheumatology, Ohta Nishinouchi General Hospital Foundation, Koriyama, Japan

**Keywords:** rheumatoid arthritis, tofacitinib, baricitinib, JAK inhibitor, IL-6 inhibitor, malignancy, major adverse cardiovascular events

## Abstract

**Background:**

The ORAL Surveillance trial showed a potentially higher incidence of malignancy and major adverse cardiovascular events (MACEs) associated with tofacitinib than those associated with tumor necrosis factor (TNF) inhibitors (TNFis). However, few studies have compared the safety of non-TNFis or other Janus kinase (JAK) inhibitors (JAKis). This study was aimed at comparing the incidence rates (IRs) of malignancies and MACEs in patients with rheumatoid arthritis (RA) treated using interleukin-6 (IL-6) inhibitors (IL-6is) or JAKis.

**Methods:**

We retrospectively analyzed 427 patients with RA who were treated using an IL-6i (n = 273) or a JAKi (n = 154). We determined the IRs of malignancy and MACEs, and the standardized incidence ratio (SIR) of malignancies and investigated factors related to malignancy and MACEs. After adjusting the clinical characteristic imbalance by propensity score matching (PSM), we compared the IRs of adverse events between the JAKi and IL-6i groups.

**Results:**

After PSM, the observational period was determined to be 605.27 patient-years (PY), and the median observational period was determined to be 2.28 years. We identified seven cases of malignancy (IR: 2.94 per 100 PY) in the JAKi-treated group and five cases (IR: 1.36 per 100 PY) in the IL-6i-treated group after PSM. The IR of MACEs was 2.56 and 0.83 (per 100 PY) in the JAKi- and IL-6i-treated groups. The IRRs of JAKi-treated patients versus IL-6i-treated patients were 2.13 (95% confidence interval (CI): 0.67–7.42) for malignancy and 3.03 (95% CI: 0.77–15.21) for MACE. There were no significant differences in IRR for malignancy and MACE between both groups after PSM. Univariate and multivariable Cox regression analyses revealed that older age and JAKi use were independent risk factors for malignancy, while older age, hypertension, and JAKi use were independent risk factors for MACEs. The overall malignancy SIR was significantly higher in the JAKi-treated group compared to the general population (2.10/100 PY, 95% CI: 1.23–2.97).

**Conclusion:**

The IRs of malignancy and MACE in patients with RA after PSM were comparable between IL-6i-treated and JAKi-treated patients. However, the SIR of malignancy in JAKi treatment was significantly higher than in the general population; therefore, further safety studies comparing JAKi to non-TNFi biologic disease-modifying antirheumatic drugs (bDMARDs) are needed.

## 1 Introduction

Rheumatoid arthritis (RA) is the most common type of autoimmune arthritis; it causes marked inflammation of the joint cartilage and bone damage and affects various other organs (1). The use of disease-modifying antirheumatic drugs (DMARDs), particularly biologic DMARDs (bDMARDs) and targeted synthetic DMARDs (tsDMARDs), such as Janus kinase (JAK) inhibitors (JAKis), theoretically enables remission to be the therapeutic goal in all patients with RA. In addition, these drugs can prevent the long-term progression of joint damage and physical dysfunction (2).

The JAK/signal transducer and activator of transcription (STAT) pathway is involved in the signal transduction of several cytokine receptors (3). JAKis inhibit the JAK-STAT pathway, leading to the inhibition of interleukin (IL)-6 and various other cytokines (4). In Japan, five JAKis have been approved for the treatment of RA: tofacitinib, baricitinib, peficitinib, upadacitinib, and filgotinib. Each JAKi has a different selectivity for JAK against each JAK isoform (5). The clinical efficacy of JAKis has been well established in large randomized controlled trials (RCTs) (6); however, there are concerns regarding the risk of adverse events (7). Recently, the ORAL Surveillance trial provided important data on the comparative safety of tofacitinib and tumor necrosis factor (TNF) inhibitors (TNFis) in the treatment of RA, raising concerns about the occurrence of malignancies and major adverse cardiovascular events (MACEs) during JAKi treatment (8). However, little is known about the comparative safety of other non-TNFi bDMARDs or JAKis other than tofacitinib. Furthermore, in the real world, JAKis tend to be introduced in patients who cannot tolerate methotrexate (MTX) because of comorbidities or in whom multiple bDMARDs have failed, which is quite different from those recruited in RCTs (9). Therefore, it would be of great interest to investigate the factors affecting MACEs and malignancy incidence of non-TNFis and JAKis in patients with RA in real-world clinical practice. We conducted the present multicenter cohort study to determine and compare the incidence rates (IRs) of MACEs and malignancies in RA patients treated with an interleukin-6 inhibitor (IL-6i) or a JAKi in clinical settings.

## 2 Materials and methods

### 2.1 Patients and study design

A multicenter retrospective cohort study was conducted to evaluate the IRs of MACEs and malignancies in patients with RA treated using an IL-6i or a JAKi. The cohort consisted of patients treated at the Department of Rheumatology of Fukushima Medical University Hospital, Japanese Red Cross Fukushima Hospital, and Ohta Nishinouchi Hospital. Between April 2012 and December 2022, IL-6i or JAKi therapy was initiated in 449 patients with RA. Among these patients, 430 started receiving IL-6i or JAKi therapy in our institution, and 427 patients for whom sufficient clinical data were available were enrolled in this study. All the patients were diagnosed with RA according to the 2010 American College of Rheumatology/European League Against Rheumatism classification criteria for RA (10). The demographic data recorded at the start of each patient’s IL-6i or JAKi treatment included age, sex, disease duration, rheumatoid factor (RF), anti-citrullinated protein antibodies (ACPAs), history of bDMARD use, coexistence of diabetes mellitus (DM) or lung disease, history of malignancy, and concomitant medication(s). The IL-6i-treated patients received tocilizumab by intravenous infusion at 8 mg/kg every 4 weeks or by subcutaneous injection of 162 mg every 2 weeks or sarilumab by subcutaneous injection of 200 mg every 2 weeks. The JAKi-treated patients received baricitinib 2 mg (in patients with renal impairment) or 4 mg once daily, tofacitinib 5 mg twice or once daily (in patients with liver impairment), upadacitinib 15 mg once daily, and filgotinib 100 mg (in patients with renal impairment) or 200 mg once daily. The study was approved by the institutional review boards of Fukushima Medical University (No. 2019-097), Japanese Red Cross Fukushima Hospital (No. 55), and Ohta Nishinouchi Hospital (No. 2022-8). An opt-out strategy was chosen for the participants, and those who declined to provide informed consent were excluded.

### 2.2 Definitions of exposure and outcomes

“Exposure” was defined as the period from the initiation of IL-6i or JAKi treatment until treatment’s discontinuation or the patient’s transfer to another hospital, death, or the end of the study period, whichever occurred first. MACEs and malignancies were focused on as adverse events. These adverse events were identified as follows. “MACEs” were defined as a composite of cardiovascular death, non-fatal myocardial infarction, and non-fatal stroke. They were determined by the patient’s attending rheumatologist or physician treating the MACE. A malignancy was defined as a composite of cancers excluding non-melanoma skin cancers. Malignancies were confirmed by the attending rheumatologist or physician treating them. All malignancies were confirmed via histological examination. Recurrent or metastatic malignancies that occurred within 1 month of the initiation of IL-6i or JAKi treatment were excluded from the analyses. The censoring time of the above-described adverse events was defined as the time from the administration of the first dose of JAKi or IL-6i until the end of the drug treatment or the last observation point, i.e., December 31, 2022.

### 2.3 Statistical analysis

Data are presented as medians and interquartile ranges for continuous variables and as frequencies and percentages for qualitative variables. The Mann–Whitney U test was used to compare continuous variables, and Fisher’s exact test was used to compare qualitative variables, as appropriate. Statistical significance for all tests was defined as a two-tailed *p*-value of <0.05. By propensity score matching in the JAKi-treated and IL-6i-treated groups, the following were analyzed: patient age; sex; disease duration; RF and ACPA positivity; MTX, glucocorticoid (GC), and b/ts DMARD use; comorbid lung disease, hypertension, and DM; and a history of malignancy. The number of adverse events, patient-years (PY) at risk, and IR ratio (IRR) with a 95% confidence interval (CI) were determined for each outcome. The time to malignancy development and MACEs in the IL-6i-treated and JAKi-treated groups were estimated using the Kaplan–Meier analysis, and log-rank tests were used to compare the cumulative IRs between the patient groups. The standardized incidence ratio (SIR) for overall malignancy (excluding cancer *in situ*) was calculated using the indirect standardization method. The estimated IRs of malignancy were determined in the general Japanese population in Fukushima Prefecture in 2019, stratified by sex and age, as reported by the Center for Cancer Control and Information Service, National Cancer Center, Japan (https://ganjoho.jp/reg_stat/statistics/data/dl/index.html). Univariate and multivariate Cox regression analyses were performed to identify the factors related to the incidences of malignancy and MACEs. Variables with *p*-values of <0.05 in univariate Cox regression analysis were included in the multivariate Cox regression analysis. Statistical analyses were performed using SPSS Statistics software (version 25.0; IBM Corp., Armonk, NY, USA) and R ver. 4.1.2 (R Foundation for Statistical Computing, Vienna, Austria, http://www.R-project.org/[accessed May 15, 2023]).

## 3 Results

### 3.1 Patients’ baseline characteristics

**Figure 1:**
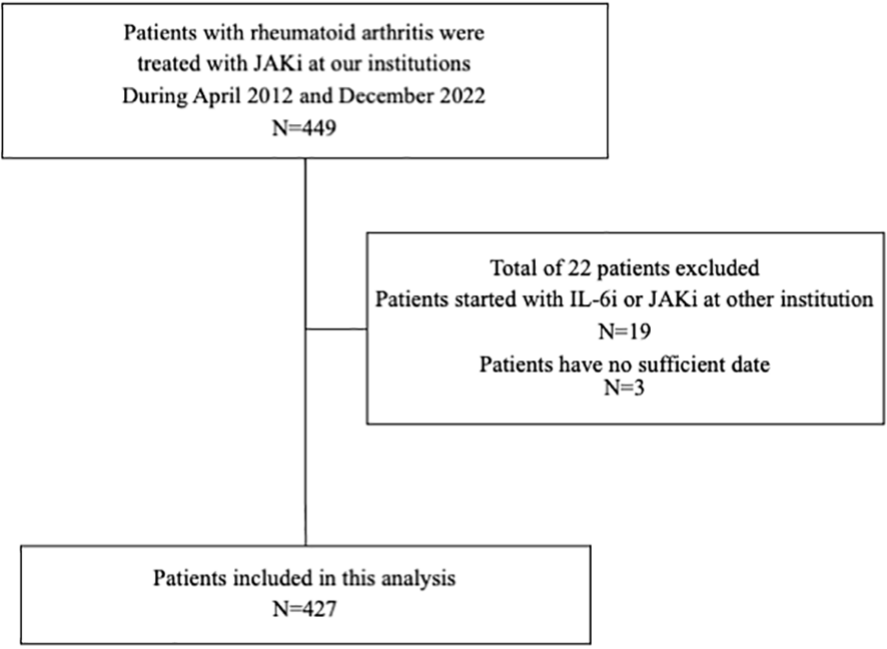
Flowchart showing patient selection. Among the 449 patients with RA who were initially treated with IL-6is or JAKis at our institution between April 2012 and December 2022, 427 for whom sufficient clinical data were available were enrolled in this study. RA, rheumatoid arthritis; IL-6i, interleukin-6 inhibitor; JAKi, Janus kinase inhibitor.

Among the 449 patients with RA in whom IL-6i or JAKi treatment was started at our institutions between April 2012 and December 2022, 427 were enrolled in this study (**Figure 1**). The background characteristics of the patients in the IL-6i-treated and JAKi-treated groups before and after the propensity score matching are summarized in **Table 1**. The baseline demographic and clinical characteristics of each patient treated with a JAKi (baricitinib, tofacitinib, upadacitinib, and filgotinib) are summarized in **Table 2**. The 273 patients in the IL-6i-treated group included 269 and 4 patients who received tocilizumab and sarilumab, respectively, and the 154 patients in the JAKi-treated group included 94, 43, 13, and 5 patients receiving baricitinib, tofacitinib, upadacitinib, and filgotinib, respectively. None of the patients had a history of using JAKi. A comparison of the two groups before propensity score weighting showed a significantly longer disease duration, higher rate of concomitant GC use, higher MTX dose, and longer observation period in the IL-6i-treated group. In contrast, the age at b/ts DMARD introduction, GC dose, and rates of hypertension and DM coexistence were significantly higher in the JAKi-treated group. The observation period for the 427 patients (135 male and 292 female patients) examined in this study was 1,264.95 PY. The median (interquartile range) length of the observation period was 2.33 (1.08–3.92) years. After propensity score matching, 220 patients with RA (70 men and 150 women) were observed for 605.27 PY. The median (interquartile range) length of the observation period was 2.28 (1.06–3.84) years. There were no significant intergroup differences after propensity score weighting, except for in the history of b/ts DMARD use and observation period. In the JAKi-treated group, malignancies and MACEs occurred only in patients treated with baricitinib and tofacitinib.

**Table 1:** Comparisons of clinical features between IL-6i group and JAKi group.

Characteristics	All patients			Propensity-matched patients			
	IL-6i (n = 273)	JAKi (n = 154)	*p*-Value	IL-6i (n = 110)	JAKi (n = 110)	*p*-Value	SMD
Male, n (%)	88 (32.2)	47 (30.5)	0.71	35 (31.8)	35 (31.8)	1.00	<0.001
Age at b/ts DMARD introduction, † years	61 (51–69)	72 (65–82)	<0.001^*^	67 (59.3–74)	68 (60–74)	0.436	0.058
Disease duration, † years	8.2 (3.5–14.9)	5.1 (1.2–13.0)	<0.001^*^	8.5 (3.7–14.0)	7.1 (2.1–15.7)	0.359	0.003
Stage, I/II/III/IV	100/76/35/45 No data, 17	58/39/16/33 No data, 8		35/34/14/22 No data, 5	45/25/9/28 No data, 3		
Class, I/II/III/IV	28/163/59/8 No data, 15	17/87/44/4 No data, 2		14/64/23/4 No data, 5	9/63/34/2 No data, 2		
RF positivity, n (%)	199 (72.9)	104 (67.5)	0.24	79 (71.8)	74 (67.3)	0.558	0.099
ACPA positivity, n (%)	204 (74.5)	108 (70.1) No data, 2	0.29	84 (76.4)	78 (70.9)	0.444	0.095
Concomitant GC use, n (%)	130 (47.6)	38 (24.7)	<0.001^*^	29 (26.4)	35 (31.8)	0.458	0.120
Concomitant GC dose, † mg/day	0.5 (0–5.0)	2.5 (0–7.0)	<0.001^*^	0 (0–3.0)	0 (0–1.75)	0.939	0.004
Concomitant MTX use, n (%)	143 (52.4)	68 (44.2)	0.10	53 (48.2)	58 (52.7)	0.590	0.091
Concomitant MTX dose, † mg/week	4 (0–8)	0 (0–6)	0.017^*^	0 (0–8.0)	4.0 (0–6.0)	0.890	0.045
Coexisting ILD, n (%)	36 (13.2)	20 (13.0)	0.95	14 (12.7)	14 (12.7)	1.00	<0.001
Coexisting hypertension, n (%)	59 (21.6)	47 (30.5)	0.041^*^	33 (30.0)	28 (25.5)	0.547	0.102
Coexisting DM, n (%)	25 (9.2)	25 (16.2)	0.029^*^	12 (10.9)	13 (11.8)	1.00	0.029
Previous history of malignancy, n (%)	24 (8.8)	14 (9.1)	0.92	9 (8.2)	9 (8.2)	1.00	<0.001
Previous use of b/ts DMARDs, n (%)	110 (67.5)	76 (49.4)	0.15	40 (36.4)	57 (51.8)	0.030^*^	0.315
Observation period,† years	2.6 (1.1–4.6)	1.7 (1.0–3.2)	<0.001^*^	2.7 (1.2–4.5)	1.7 (1.0–3.3)	0.005^*^	0.516

IL-6i, interleukin-6 receptor inhibitors; JAKi, Janus kinase inhibitors; RF, rheumatoid factor; ACPA, anti-citrullinated peptide antibody; GC, glucocorticoid; MTX, methotrexate; ILD, interstitial lung disease; DM, diabetes mellitus; b/ts DMARD, biologic/targeted synthetic disease-modifying antirheumatic drug; SMD, standardized mean difference.

† Values are the median with interquartile range.

^*^ There is a significant difference at p < 0.05.

**Table 2:** Comparisons of clinical features of each JAKi.

	Baricitinib (n = 94)	Tofacitinib (n = 43)	Upadacitinib (n = 12)	Filgotinib (n = 5)
Male, n (%)	25 (26.6)	12 (27.9)	7 (58.3)	3 (60.0)
Age at b/ts DMARD introduction, † years	74 (67–84)	72 (66–79)	59 (54–66)	61 (61–65)
Disease duration, † years	4.8 (0.6–14.0)	5.39 (2.0–12.1)	4.7 (2.7–8.4)	7.0 (6.4–10.4)
Stage, I/II/III/IV	36/20/12/20 No data, 6	18/13/1/9 No data, 2	3/5/2/2	1/1/1/2
Class, I/II/III/IV	15/52/23/3 No data, 1	2/21/18/1 No data, 1	0/9/3/0	0/5/0/0
RF positivity, n (%)	64 (68.1)	26 (60.5)	10 (83.3)	4 (80.0)
ACPA positivity, n (%)	67 (71.3) No data, 1	26 (60.5) No data, 1	11 (91.7)	4 (80.0)
Concomitant GC use, n (%)	17 (18.1)	11 (25.6)	8 (66.7)	2 (40.0)
Concomitant GC dose, † mg/day	0.0 (0.0–0.0)	0.0 (0.0–1.75)	2.0 (0.0–2.5)	0.0 (0.0–5.0)
Concomitant MTX use, n (%)	35 (37.3)	27 (62.8)	4 (33.3)	3 (60.0)
Concomitant MTX dose, † mg/week	0.0 (0.0–6.0)	4.0 (0.0–7.0)	0.0 (0.0–4.0)	6.0 (0.0–6.0)
Coexisting ILD, n (%)	12 (12.8)	6 (14.0)	2 (16.7)	0
Coexisting hypertension, n (%)	30 (31.9)	14 (32.6)	3 (25.0)	0
Coexisting DM, n (%)	15 (16.0)	9 (20.9)	1 (8.3)	0
Previous history of malignancy, n (%)	11 (11.7)	2 (4.7)	1 (8.3)	0
Previous use of b/ts DMARDs, n (%)	44 (46.8)	23 (53.5)	8 (66.7)	1 (20.0)
Observation period,† years	1.77 (1.16–3.21)	2.08 (1.02–3.57)	1.3 (1.1–1.4)	0.6 (0.6–0.7)
Malignancy	5 (5.3)	7 (16.3)	0	0
MACEs	5 (5.3)	5 (11.6)	0	0

RF, rheumatoid factor; ACPA, anti-citrullinated peptide antibody; GC, glucocorticoid; MTX, methotrexate; ILD, interstitial lung disease; DM, diabetes mellitus; b/ts DMARD, biologic/targeted synthetic disease-modifying antirheumatic drug; MACEs, major adverse cardiovascular events.

† Values are the median with interquartile range.

### 3.2 IR of malignancy

**Figure 2:**
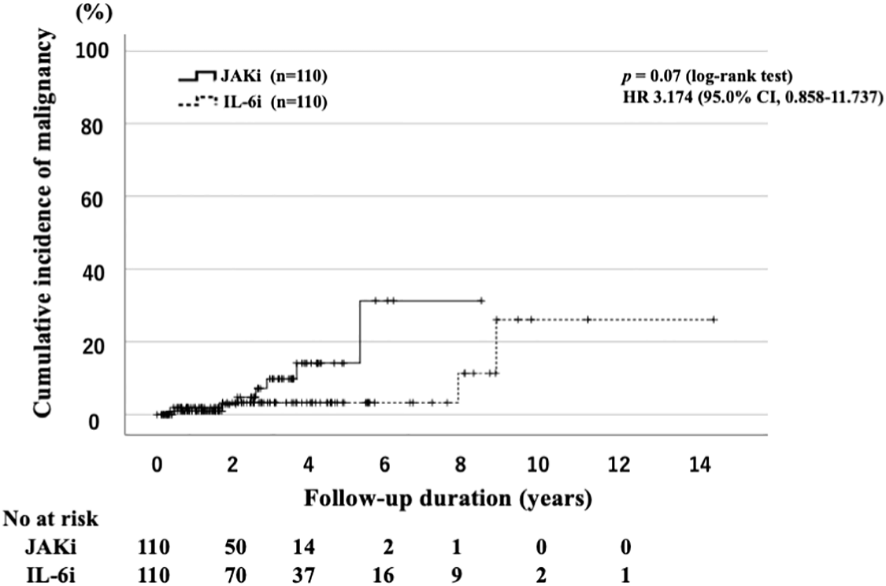
Cumulative incidence curves of malignancy in IL-6i-treated and JAKi-treated patients after propensity score matching. Kaplan–Meier curves showing the cumulative incidence of malignancies in patients treated with IL-6is (n = 110) and JAKis (n = 110). No significant differences were observed between IL-6i-treated and JAKi-treated groups. The starting point (0 years) was the date on which the observations began. RA, rheumatoid arthritis; IL-6i, interleukin-6 inhibitor; JAKi, Janus kinase inhibitor; No, number.

The IRs for malignancies are shown in **Table 3**. We identified 12 cases of malignancy (7.8%; IR: 3.70 per 100 PY; number needed to harm [NNH]: 27.06 PY) among 154 JAKi-treated patients. The most frequent malignancy in the JAKi-treated group was lymphoma (n = 6; 50% of all malignancies). The other six malignancies in the JAKi-treated group were lung cancer (n = 2), rectal cancer (n = 2), colon cancer (n = 1), and malignant melanoma (n = 1). Among the 273 IL-6i-treated patients, 10 had malignancies (3.7%; IR: 1.06 per 100 PY; NNH: 94.03 PY). In the IL-6i-treated group, the most frequent malignancy was lung cancer (n = 3; 30% of all malignancies). The other seven malignancies were colon cancer (n = 2), malignant lymphoma (n = 1), breast cancer (n = 1), pancreatic cancer (n = 1), prostate cancer (n = 1), and ovarian cancer (n = 1). Before propensity score weighting, the IRR for the JAKi-treated group to the IL-6i-treated group was 3.41, indicating that the JAKi-treated group was more likely to develop a malignancy than the IL-6i-treated group (95% CI: 1.48–8.30, p = 0.005). However, after propensity score weighting, there was no significant difference in the IRRs between the two groups (IRR = 2.13, 95% CI: 0.67–7.42, p = 0.20). The follow-up period of patients treated with JAKi was shorter than that of patients treated with IL-6i. Therefore, we evaluated the time-to-event outcome (malignancy) using the Kaplan–Meier curves. There was no significant difference in the cumulative incidence of malignancy between the groups (**Figure 2**). A comparison of the incidence of malignancy between the baricitinib and tofacitinib (JAKis) groups is shown in **Table 4**. The crude IR of malignancy was higher in the tofacitinib group than in the baricitinib group. There was no significant difference in the IRRs between the baricitinib and tofacitinib groups (IRR = 2.46, 95% CI: 0.77–8.45, p = 0.13). Compared with the general Japanese population, the SIR for all malignancies in JAKi treatment (2.10, 95% CI: 1.23–2.97) was significantly higher, and the SIR in IL-6i treatment (1.09, 95% CI: 0.56–1.61) was comparable (**Table 5**).

**Table 3:** Comparisons of Incidence rate of malignancy and MACE between JAKi group and IL-6i group.

	All patients			Propensity-matched patients		
	IL-6i (n = 273)	JAKi (n = 154)	*p*-Value	IL-6i (n = 110)	JAKi (n = 110)	*p*-Value
Malignancy	10 (3.7)	12 (7.8)		5 (4.5)	7 (6.4)	
IR per 100 PY (95% CI)	1.06 (0.41–1.71)	3.70 (1.65–5.75)		1.36 (0.18–2.54)	2.94 (0.80–5.08)	
IRR (95% CI)	1 [reference]	3.47 (1.48–8.30)	0.005^*^	1 [reference]	2.13 (0.67–7.42)	0.20
MACEs	4 (1.5)	10 (6.5)		3 (2.7)	6 (5.5)	
IR per 100 PY (95% CI)	0.43 (0.02–0.84)	3.08 (1.2–4.96)		0.83 (–0.1–1.76)	2.56 (0.54–4.58)	
IRR (95% CI)	1 [reference]	7.07 (2.33–26.63)	<0.001^*^	1 [reference]	3.03 (0.77–15.21)	0.11

MACEs, major adverse cardiovascular events; IR, incidence rate; IRR, incidence rate ratio; PY, patient-years; CI, confidence interval.

**Table 4:** Comparisons of the incidence rate of malignancy and MACE between patients treated with baricitinib and tofacitinib.

	Baricitinib (n = 94)	Tofacitinib (n = 43)	*p*-Value
Malignancy	5 (5.3%)	7 (16.3%)	
IR per 100 PY (95% CI)	2.53 (0.34–4.72)	6.48 (1.84–11.12)	
IRR (95% CI)	1 [reference]	2.46 (0.77–8.54)	0.13
MACE	5 (5.3%)	5 (11.6%)	
IR per 100 PY (95% CI)	2.53 (0.34–4.72)	4.54 (0.65–8.43)	
IRR (95% CI)	1 [reference]	1.79 (0.48–6.66)	0.37

MACEs, major adverse cardiovascular events; IR, incidence rate; IRR, incidence rate ratio; PY, patient-years; CI, confidence interval.

**Table 5:** Observed and expected numbers of malignancies and their SIR with 95% CI.

	O/E	SIR	95% CI	*p*-Value
JAKi	12/5.71	2.10	1.23–2.97	0.02^*^
IL-6i	10/9.21	1.09	0.56–1.61	0.76

IL-6i, interleukin-6 receptor inhibitors; JAKi, Janus kinase inhibitors; CI, confidence interval; E, expected number; O, observed number; SIR, standardized incidence ratio.

### 3.3 IR of MACEs

**Figure 3:**
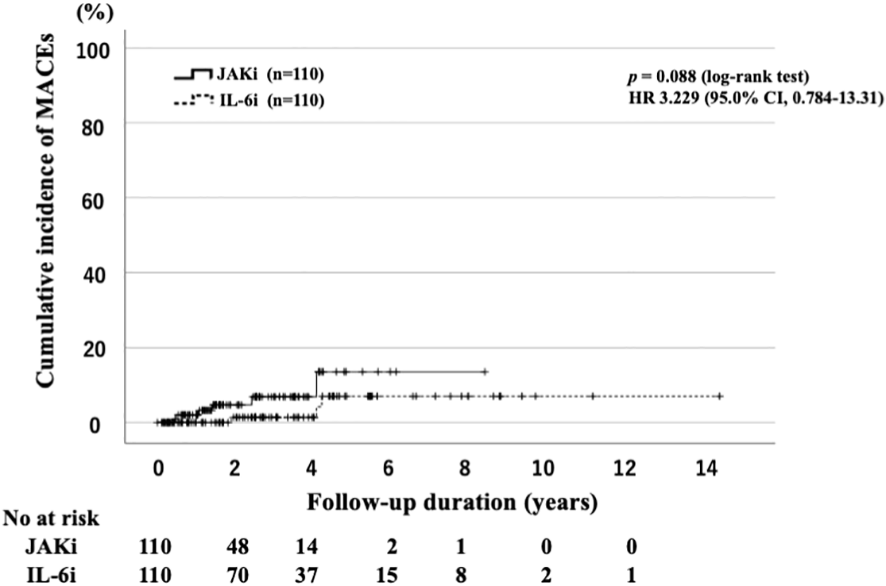
Cumulative incidence curves of MACEs in IL-6i-treated and JAKi-treated patients after propensity score matching. Kaplan–Meier curves showing the cumulative incidence of MACEs in patients treated with IL-6is (n = 110) and JAKis (n = 110). No significant differences were observed between IL-6i-treated and JAKi-treated groups. The starting point (0 years) was the date on which the observations began. RA, rheumatoid arthritis; IL-6i, interleukin-6 inhibitor; JAKi, Janus kinase inhibitor; MACEs, major cardiovascular events; No, number.

The IRs of MACEs are listed in **Table 3**. We determined the IR of MACEs and identified 10 cases of MACEs (6.5%; IR: 3.08 per 100 PY; NNH: 32.13 PY) in the JAKi-treated group. The MACEs in the JAKi-treated group included acute cardiac insufficiency (n = 4), brain hemorrhage (n = 2), subarachnoid hemorrhage (n = 2), acute myocardial infarction (n = 1), and aortic dissection (n = 1). There were four cases of MACEs in the IL-6i-treated group (1.5%; IR: 0.43 per 100 PY; NNH: 233.98 PY). In the IL-6i-treated group, the four MACEs were aortic dissection (n = 2), acute myocardial infarction (n = 1), and acute cardiac insufficiency (n = 1). Before the propensity score weighting, the IRR for the JAKi-treated group to the IL-6i-treated group was 7.07, indicating that the JAKi-treated group is more likely to occur MACEs than the IL-6i-treated group (95% CI: 2.33–26.63, *p* < 0.001). However, after propensity score weighting, there was no significant difference in the incidence of MACEs between the JAKi-treated and IL-6i-treated groups (IRR = 3.03, 95% CI: 0.77–15.21, *p* = 0.11). Furthermore, there was no significant difference in the cumulative incidence of MACE between the JAKi-treated and IL-6i-treated groups (**Figure 3**). A comparison of the incidence of MACEs between the baricitinib and tofacitinib groups is shown in **Table 4**. The crude IRs of MACEs were higher in the tofacitinib group than in the baricitinib group. There was no significant difference in the IRRs between the baricitinib and tofacitinib groups (IRR = 1.79, 95% CI: 0.48–6.66, *p* = 0.37).

### 3.4 Factors related to malignancy and MACEs in b/ts DMARD treatment

To identify the risk factors associated with malignancy and MACEs, we analyzed the baseline characteristics of the patients by using univariate and multivariate Cox regression analyses (**Table 6**). Univariate analysis indicated that age of >65 years and JAKi use were associated with the incidence of malignancy. Multivariate analysis showed similar results. Univariate analysis showed that age of >65 years, coexisting hypertension, and JAKi use were associated with MACEs.

**Table 6:** Independent risk factors for malignancy and MACE. .

Risk factors for malignancy				
Variable	Univariate model		Multivariate model	
	HR (95% CI)	*p*-Value	HR (95% CI)	*p*-Value
Age, >65 years or not	5.033 (1.826–13.878)	0.002^*^	3.658 (1.231–10.869)	0.020^*^
Disease duration, per 1-year increase	1.006 (0.960–1.054)	0.790		
RF positive or negative	1.076 (0.420–2.755)	0.879		
ACPA positive or negative	1.152 (0.423–3.137)	0.781		
GC use, yes/no	1.153 (0.492–2.700)	0.348		
GC dose, per 1-mg increase	1.044 (0.954–1.143)	0.345		
MTX use, yes/no	0.559 (0.234–1.336)	0.191		
MTX dose, per 1-mg increase	0.933 (0.831–1.047)	0.236		
Coexisting DM, yes/no	1.625 (0.476–5.545)	0.438		
Previous history of malignancy, yes/no	1.081 (0.252–4.636)	0.917		
No. of previous use of b/ts DMARDs, per drug	1.470 (0.999–2.163)	0.051		
JAKi use, yes/no	5.370 (2.026–14.276)	<0.001^*^	3.754 (1.348–10.498)	0.012^*^
Risk factors for MACEs				
Variable	Univariate model			
	HR (95% CI)		*p*-Value	
Age, >65 years or not	7.714 (1.706–34.887)		0.008^*^	
Disease duration, per 1-year increase	0.925 (0.848–1.010)		0.081	
RF positive or negative	1.034 (0.324–3.301)		0.955	
ACPA positive or negative	1.193 (0.328–4.344)		0.789	
GC use, yes/no	0.904 (0.302–2.702)		0.856	
GC dose, per 1-mg increase	0.925 (0.775–1.106)		0.393	
MTX use, yes/no	0.940 (0.328–2.688)		0.907	
MTX dose, per 1-mg increase	0.924 (0.799–1.069)		0.289	
Coexisting HT, yes/no	5.929 (1.983–17.728)		0.001^*^	
Coexisting DM, yes/no	2.582 (0.715–9.322)		0.148	
JAKi use, yes/no	9.616 (2.779–33.273)		<0.001^*^	

HR, hazard ratio; CI, confidence interval; RF, rheumatoid factor; ACPA, anti-citrullinated peptide antibody; GC, glucocorticoid; MTX, methotrexate; ILD, interstitial lung disease; DM, diabetes mellitus; JAKi, Janus kinase inhibitors; b/ts DMARD, biologic/targeted synthetic disease-modifying antirheumatic drug; MACEs, major adverse cardiovascular events.

^*^ There is a significant difference at p < 0.05.

## 4 Discussion

The ORAL Surveillance trial provided important data concerning the safety of JAKis, showing an increased risk of MACEs and malignancy in patients with RA treated using tofacitinib compared with those treated using adalimumab among patients with cardiovascular risk factors (8). Studies have identified several risk factors for MACEs and cancer, including older age (>65 years), smoking, and a history of venous thromboembolism, MACEs, or cancer (11). The Food and Drug Administration issued warnings regarding the increased risk of MACEs and malignancy in patients with RA treated using JAKis (12). Following the ORAL Surveillance trial, several studies have been conducted to evaluate the safety of JAKis compared with TNFis in real-world patients with RA (13, 14). However, most of these studies found no evidence of an increased risk of cancer other than non-melanoma skin cancer in patients treated with JAKis compared with those with TNFis (13, 14). A recent meta-analysis also showed that JAKi treatment in real-world patients with RA was not associated with a significantly increased risk of the first primary cancer compared with those who received bDMARDs (15). However, few studies have comparatively assessed the safety of other non-TNFi bDMARDs or JAKi bDMARDs other than tofacitinib. We aimed to compare the incidence of cancer and MACEs in patients with RA treated with tocilizumab or JAKis in a real-world setting. Despite the different baseline characteristics, our data demonstrated that patients with RA treated using JAKis had a higher risk of developing MACEs and cancer than those treated with IL-6is. However, in the propensity score matching analysis, we did not observe any increase in the overall occurrence of cancer with JAKis compared to that with IL-6is. The follow-up period of patients with RA treated using JAKis was shorter than that of those treated with IL-6is. We evaluated the time-to-event outcomes (cancer and MACEs) using the Kaplan–Meier curves to minimize the influence of differences in follow-up time; however, there was no significant difference between these two groups, and the occurrence of malignancies in either the IL-6i-treated or TNFi-treated patients was generally infrequent in commercial databases with an IR of less than 10/1,000 PY (16). In our study, the IR for malignancy was 1.06/100 PY in patients with RA treated using IL-6is. Our results are consistent with those of previous studies (16).

In the ORAL Surveillance trial, the IR for malignancy was 1.13 (95% CI: 0.86–1.14) for those treated with tofacitinib 10 mg twice daily, compared with 0.77 among those treated using TNFis (95% CI: 0.55–1.04), indicating the significant risk associated with JAKis (hazard ratio (HR): 1.48; 95% CI: 1.04–2.09) (8). However, in RCTs or long-term extension studies, the overall rate of malignancy for JAKis has been reported to be similar to that associated with bDMARDs (17, 18). A recent multi-database cohort study also found no difference in the risk of malignancies, excluding non-melanoma skin cancer (NMSC), in patients with RA treated using tocilizumab compared with those treated with TNFis (19, 20). In our propensity score matching analysis, cancer IRs were similar between patients with RA treated using JAKis and IL-6is. Although the difference was not significant, the IR of malignancy in patients with RA treated using JAKis was 2.94 (95% CI: 0.80–5.08), which was higher than that in the previous studies and suggests the need for further safety assessments (14). Concerning risk factors, elderly age and the use of JAKis were identified as independent risk factors for malignancies in our study. This finding is consistent with the findings of earlier reports (11). Patients with RA are considered to have an increased risk of cancer, including lymphoma, compared with the general population (21, 22). The incidence of lymphoma was higher (6/12) in patients treated with JAKis than in those treated with tocilizumab (1/10). The mechanism by which JAKis are associated with some types of cancer is unknown; however, it can be speculated that JAKis may affect the functions of natural killer cells, which could potentially diminish the immunosurveillance of the host for cancer. Nevertheless, we could not exclude residual or unmeasured confounding factors in our propensity score matching comparisons between JAKis and tocilizumab. Further monitoring of the safety of JAKis and other bDMARDs is warranted because there are ongoing safety concerns about MACEs in patients with RA treated using JAKis.

In the ORAL Surveillance trial, which included patients with active RA aged >50 years and with a least one cardiovascular risk factor, the results indicated that the incidence of MACEs associated with tofacitinib was higher than that associated with TNFis (HR: 1.33; 95% CI: 0.91–1.94) (8). A *post-hoc* analysis showed a higher MACE risk with tofacitinib than with TNFis in patients with RA and a history of atherosclerotic cardiovascular disease (23). By contrast, no clear difference was observed in the risk of MACEs among patients without a history of atherosclerotic cardiovascular disease (23). In the propensity score matching analysis, the IRs of MACEs were similar between patients with RA treated using JAKis and those treated using IL-6is in our study. Although the difference was not significant, in patients treated with JAKis, the IR of MACEs was relatively high, which requires further safety assessment. Factors such as older patient age and the presence of DM were considered risk factors for MACEs in patients with RA in our study. The patients in the ORAL Surveillance trial were aged >50 years and had at least one additional cardiovascular risk factor, whereas the elderly patients in the present study had varying backgrounds, which might explain why MACEs occurred frequently in our cohort. Several studies have indicated the possibility of an increased incidence of MACEs in patients treated with JAKis (23, 24). Concerning the risk for MACEs, factors such as an advanced age (>65 years) and concomitant GC treatment were described in earlier reports (23, 24). More elderly patients with RA at risk for MACEs and cancer were enrolled in this study than in previous studies (8); this might explain why more MACEs occurred in our study.

Our study had several limitations. The number of patients (n = 427) and the duration of the follow-up period (April 2012 through December 2022) were not sufficient to detect all adverse events. Furthermore, the median follow-up period for the patients after propensity score matching was only 2.3 years, despite the 10-year recruitment period. The follow-up period was shorter in the JAKi group than in the IL-6i group, and that may affect the fewer adverse events in the JAKi group. Therefore, this study may not be adequately powered to determine the risk of malignancies or MACEs associated with the long-term use of JAKis or bDMARDs. Malignancy and MACE are rare outcomes, and not all factors that were significantly different in univariate Cox regression analysis could be included in the multivariate analysis. The choice of treatment was made at the discretion of each rheumatologist with no standardized protocol. In addition, the JAKi used in this study was not limited to the same agent. We also did not have data on family history of cancer, body mass index, smoking, and alcohol intake. Some patients with RA receive more than one b/ts DMARD, and previous b/ts DMARD exposure may influence the risk of cancer or MACEs. Adverse events, such as MACEs, may be affected by disease activity (25); however, the precise disease activity was not evaluated in this study.

In conclusion, there was no significant difference in the incidences of cancer and MACEs between patients with RA treated using JAKis and IL-6is in our propensity-matched analysis with a retrospective cohort design. The SIR of malignancy in JAKi treatment was significantly higher than in the general population. Although the difference was not significant, the IRs of MACEs and cancer seemed higher in patients with RA treated using JAKis than in those treated using IL-6is, suggesting the need for more safety studies comparing JAKis and non-TNFi bDMARDs.

## Data availability statement

The raw data supporting the conclusions of this article will be made available by the authors, without undue reservation.

## Ethics statement

The studies involving humans were approved by the institutional review boards of Fukushima Medical University (No. 2019-097), Japanese Red Cross Fukushima Hospital (No. 55), and Ohta Nishinouchi Hospital (No. 2022–8). The studies were conducted in accordance with the local legislation and institutional requirements. The ethics committee/institutional review board waived the requirement of written informed consent for participation from the participants or the participants’ legal guardians/next of kin because an opt-out strategy was chosen for the participants, and those who declined to provide informed consent were excluded.

## Author contributions

SY: Conceptualization, Data curation, Formal Analysis, Methodology, Project administration, Software, Validation, Visualization, Writing – original draft, Writing – review & editing. MM: Data curation, Supervision, Validation, Writing – review & editing. ES: Data curation, Supervision, Writing – review & editing, Validation. TK: Data curation, Supervision, Validation, Writing – review & editing. YS: Data curation, Validation, Writing – review & editing. KS: Data curation, Validation, Writing – review & editing. HM: Data curation, Validation, Writing – review & editing. JT: Data curation, Validation, Writing – review & editing. YF: Data curation, Validation, Writing – review & editing. NM: Data curation, Validation, Writing – review & editing. TA: Data curation, Validation, Writing – review & editing. SS: Data curation, Validation, Writing – review & editing. KM: Conceptualization, Data curation, Funding acquisition, Investigation, Methodology, Project administration, Resources, Supervision, Validation, Writing – original draft, Writing – review & editing.
